# The Evolving Story of Multifactorial Chylomicronemia Syndrome

**DOI:** 10.3389/fcvm.2022.886266

**Published:** 2022-04-14

**Authors:** Martine Paquette, Sophie Bernard

**Affiliations:** ^1^Genetic Dyslipidemias Clinic, Montreal Clinical Research Institute, Montreal, QC, Canada; ^2^Division of Endocrinology, Department of Medicine, Université de Montréal, Montreal, QC, Canada

**Keywords:** multifactorial chylomicronemia syndrome, hypertriglyceridemia, genetic, diet, pancreatitis, cardiovascular disease

## Abstract

Multifactorial chylomicronemia syndrome (MCS or type V hyperlipoproteinemia) is the most frequent cause of severe hypertriglyceridemia and is associated with an increased risk of acute pancreatitis, cardiovascular disease, and non-alcoholic steatohepatitis. The estimated prevalence of MCS in the North American population is 1:600–1:250 and is increasing due to the increasing prevalence of obesity, metabolic syndrome, and type 2 diabetes. Differentiating between familial chylomicronemia syndrome and MCS is crucial due to their very different treatments. In recent years, several cohort studies have helped to differentiate these two conditions, and recent evidence suggests that MCS itself is a heterogeneous condition. This mini-review will summarize recent literature on MCS, with a specific focus on the genetic determinants of the metabolic risk and the latest developments concerning the pharmacological and non-pharmacological treatment options for these patients. Possible research directions in this field will also be discussed.

## Introduction

Severe hypertriglyceridemia (HTG) is defined as a fasting triglycerides (TG) concentration of ≥10 mmol/L (>885 mg/dL). At this threshold of TG, a pathological accumulation of circulating chylomicrons (chylomicronemia) is almost always present in the plasma (1). Multifactorial chylomicronemia syndrome (MCS) (OMIM #144650), previously known as “type V hyperlipoproteinemia” according to the Fredrickson classification or “late-onset chylomicronaemia,” is by far the most common form of chylomicronemia and severe HTG. Although the exact frequency of MCS in the general population is not precisely known, the prevalence of severe HTG in North America has been estimated to be between 1:600 and 1:250 (2–4). In order to develop MCS, an underlying genetic susceptibility for impaired TG metabolism must be present (5). The full expression of the MCS phenotype is then triggered by the presence of secondary factors such as a diet rich in fats and simple carbohydrates, reduced activity levels, obesity, metabolic syndrome, alcohol intake, and uncontrolled diabetes (6). In these patients, both chylomicrons and very low-density lipoproteins (VLDLs) are increased in circulation due to impairment of lipoprotein lipase (LPL) activity as well as hepatic overproduction of VLDLs and their reduced clearance. Following blood sampling, centrifugation, and overnight storage at 4°C, the presence of chylomicrons can be observable if a creamy supernatant layer is present on the top of the tube, whereas a cloudy and lactescent lower layer (infranatant) indicates the presence of VLDLs (7, 8). Clinically, the main manifestations associated with MCS include the presence of eruptive xanthomas, lipemia retinalis, abdominal pain, and impaired concentration (6). Furthermore, MCS is associated with an increased risk of serious health consequences, which includes acute pancreatitis (AP), cardiovascular disease (CVD), as well as non-alcoholic fatty liver disease (NAFLD) (9).

## Complications of MCS

The risk of AP in MCS patients as compared to normolipidemic individuals from the general population is at least 7-fold higher (between ~7- and 54-fold) (7, 10, 11), whereas the risk of CVD is 2- to 9-fold higher (7, 11). The presence of TG-rich lipoprotein remnants in circulation, which can penetrate the vascular wall, as well as the concomitant presence of atherogenic comorbidities such as obesity or diabetes, could explain the increased cardiovascular risk, whereas the exact mechanisms linking HTG and AP still remain poorly understood (12–14). However, patients with HTG generally present a more severe clinical course of AP, with increased morbidity and mortality (15, 16).

NAFLD is a chronic liver disease characterized by excessive fat accumulation in the liver and is considered as the hepatic component of the metabolic syndrome or a consequence of it. The prevalence of NAFLD in the general population is estimated to be around 25% (17). NAFLD can progress to non-alcoholic steatohepatitis and ultimately to cirrhosis and its complications (18). Excessive circulating TG represents one of the risk factors associated with the development of NAFLD (19–21). In a recent study, the prevalence of NAFLD in patients affected by MCS was studied for the first time using transient elastography (FibroScan). The authors observed that the prevalence of NAFLD was 74% in 19 MCS subjects, which is three times more prevalent than in the general population (22). Interestingly, the authors observed a negative correlation between liver fat accumulation and AP risk in these patients. This may suggest that if more TG accumulates in the liver, a lower quantity would be available to contribute to the pathophysiology of AP (22). However, because of the small sample size of this study, these results need to be replicated in a larger cohort of MCS patients.

## Differences Between MCS and FCS

Familial chylomicronemia syndrome (FCS) (OMIM #238600, also known as type I hyperlipoproteinemia, LPL deficiency, or monogenic chylomicronemia) is a rare autosomal recessive disorder, also associated with severe HTG and risk of life-threatening AP. In these patients, the severe HTG in the fasting state is solely explained by the presence of chylomicrons. Compared to MCS, FCS is less common, with an estimated prevalence of 1 to 10 per million (6). Because there exists a large overlap in the phenotype of FCS and MCS, the differential diagnosis between these two conditions may be a challenge. However, making a proper diagnosis is important to guide appropriate treatment. In recent years, there has been increasing interest for studying the clinical differences between FCS and MCS patients (10, 11, 23–29). The study of Paquette et al. was the first that systematically compared the clinical and biochemical characteristics of genetically confirmed FCS patients (*n* = 25) vs. MCS patients (*n* = 36) (24). In this study, despite similar TG concentrations (19.57 mmol/L in FCS vs. 25.12 mmol/L in MCS), the severity of the disease was generally worse in FCS patients than in MCS patients, with a significantly higher prevalence of abdominal pain (63 vs. 19%), pancreatitis (60 vs. 6%), and multiple pancreatitis (48 vs. 3%). Furthermore, chylomicronemia discovery occurred at a younger age in FCS patients (11 vs. 36 years) and more frequently because of clinical complications including an episode of pancreatitis (12 vs. 3%) and the presence of abdominal pain (20 vs. 3%) than in MCS patients. In contrast, the cardiometabolic profile was better in FCS than in MCS patients. Indeed, when the number of metabolic syndrome features was studied [including body mass index (BMI) ≥ 27 kg/m^2^, systolic blood pressure ≥ 130 mmHg or diastolic blood pressure ≥ 85 mmHg (or treatment for hypertension) and fasting glucose ≥ 5.6 mmol/L (or treatment for diabetes)], a lower frequency of patients presenting two or three abnormalities was observed in FCS (10%) compared with MCS (67%). In addition, the prevalence of CVD was lower in FCS than in MCS patients (0 vs. 17%), although the difference was not statistically significant. Other significant differences between groups included lower ALT, GGT, total cholesterol, HDL-C, LDL-C, and non-HDL-C in FCS compared to MCS patients (24). Several findings of this study were confirmed in other cohorts. Indeed, the frequency of pancreatitis was observed to be higher in FCS (ranging from 59 to 88%) than in MCS (ranging from 11 to 37%) (11, 25, 26, 28, 29). In the study of Belhassen et al., which is a prospective study over 10 years of follow-up (median of 9.8 years), the hazard ratio for incident AP was 3.6 in FCS as compared with MCS (11). These results are similar to those of D'Erasmo et al., who reported an incidence rate of AP in FCS three times higher than in MCS over a median follow-up period of 44 months (25). The younger age at baseline in FCS is also frequently reported, with >10 years of difference between these two conditions (11, 27, 28). Concerning the metabolic profile of these patients, a lower BMI is always observed in FCS patients when this variable is studied (11, 26–29). Indeed, FCS patients are typically within the normal BMI range, whereas the average BMI in MCS patients is mostly between 28 and 30 kg/m^2^. A higher frequency of NAFLD has also been reported in MCS (74%) compared with FCS (42%) (22). However, conflicting results have been obtained concerning differences in prevalence or incidence of CVD, diabetes, and hypertension (11, 26, 28). Echoing the findings of Paquette et al. (24), others found significantly lower concentrations of total cholesterol (25), HDL-C (25–27), and LDL-C (26, 27) in FCS patients compared with MCS patients. Importantly, the apolipoprotein B (apoB) concentration in FCS patients was also found to be significantly lower than in MCS patients, with minimal overlap between groups (28, 30). Indeed, apoB cutoffs between 0.75 and 0.9 g/L have been proposed in order to differentiate FCS patients from MCS patients (28, 31). Finally, although TG concentrations are highly fluctuating, the majority of studies found a higher concentration of baseline TG or maximal TG in FCS subjects than in MCS subjects (11, 27–29).

## Genetics of Severe Hypertriglyceridemia

The gold standard for the differential diagnosis of FCS and MCS in patients with severe HTG remains the genetic testing using a targeted next-generation DNA sequencing panel (32). Other existing diagnostic strategies to identify FCS patients are described elsewhere (8). Whereas, the presence of homozygous or compound heterozygous rare variants in the canonical genes involved in TG metabolism (*LPL* gene or, less frequently, its modulators: *APOC2, GPIHBP1, APOA5*, and *LMF1* genes) is indicative of FCS, the molecular basis of MCS is more complex. Indeed, there are two main genetic determinants that confer susceptibility to MCS: the presence of a single deleterious rare variant in one of the five main TG genes (heterozygous) or the accumulation of several single-nucleotide polymorphisms (SNPs) associated with TG concentration (polygenic). This polygenic susceptibility to MCS is quantified using a polygenic risk score with common SNPs extracted from genome-wide association studies. Recent publications have shed light on the complex genetic architecture of this disease and on the proportion of each of the main types of genetic determinants found in the severe HTG population. In the study of Dron et al. in a cohort of 563 Caucasian patients with severe HTG, the authors found that a high polygenic risk score (comprised of 16 SNPs) was the most common genetic determinant of this trait in adults. Indeed, FCS (biallelic rare variants) was found in 1.1% of the cohort, whereas heterozygous rare variant and high polygenic risk were found in 14.4 and 32.0% of the cohort, respectively. Of note, half of the studied cohort remained genetically undefined (5). The involvement of non-canonical secondary TG genes has been suggested as one of the possible factors explaining the severe HTG in these patients with no identified genetic basis (5, 33–36). Also, multiple polygenic risk scores exist, with variable number of SNPs, which can be weighted or unweighted (37, 38). In a subsequent study from the same group, an overview of the genetic determinants of severe HTG was compared in patients from three different ancestry groups: European (*n* = 336), East Asian (*n* = 63), and Hispanic (*n* = 199). Whereas, the proportion of patients with high polygenic scores was similar between groups (frequency of 25.4–33.9%), the proportion of patients carrying deleterious rare variants [heterozygous (MCS) or biallelic (FCS)] differed. This proportion was the highest in the Hispanic cohort (36.7%), followed by the East Asian cohort (25.4%), and the European cohort (14.3%) (39). However, it should be kept in mind that TG-associated SNPs included in polygenic scores are mainly from European-based genome-wide association studies. In some isolated populations, a founder effect is present for specific deleterious rare variants, which increases the prevalence of heterozygous carriers. For example, in the French Canadian population, there exists an enrichment in two *LPL* variants [p.(Gly215Glu) and p.(Pro234Leu)]. Accordingly, the reported prevalence of MCS patients carrying a rare variant in this population is higher than expected in Caucasian patients (30, 40).

## Risk Stratification and Heterogeneity of MCS

Although there is now a better understanding of the clinical and biochemical differences between FCS and MCS patients, the phenotype heterogeneity among MCS patients remains poorly studied. In a recent publication, the clinical differences between MCS patients with (positive-MCS) vs. without (negative-MCS) a rare deleterious variant in the five canonical genes involved in TG metabolism have been studied for the first time (30). The main observation of this study is that the positive-MCS group (*n* = 22) had an intermediate phenotype severity between the FCS (*n* = 28) and negative-MCS (*n* = 53) groups. Indeed, there was a significant difference between the three groups concerning the prevalence of abdominal pain (59% in FCS, 36% in positive-MCS, and 15% in negative-MCS), pancreatitis (61% in FCS, 41% in positive-MCS, and 9% in negative-MCS), and multiple pancreatitis (46% in FCS, 23% in positive-MCS, and 6% in negative-MCS). However, when the MCS groups were compared, the age of the first pancreatitis was not different (41 years in positive-MCS and 48 years in negative-MCS), and there was no difference concerning the prevalence of CVD. Interestingly, while the baseline TG concentration was similar between positive-MCS (10.55 mmol/L) and negative-MCS (10.33 mmol/L), the maximal recorded TG value was higher in positive-MCS (41.03 mmol/L) than in negative-MCS (19.50 mmol/L). Importantly, while the lower apoB concentration in FCS patients as compared with MCS patients has been well-documented in previous studies, this study showed for the first time that the apoB value was also significantly lower in the positive-MCS group (0.80 g/L) compared to the negative-MCS group (1.10 g/L). Of note, among MCS patients, an apoB value <1 g/L was associated with a ~5-fold increased risk of pancreatitis. This cutoff was therefore suggested in order to identify higher-risk individuals among patients with severely elevated TG concentrations and to prioritize them for genetic screening. In this cohort, strong predictors of pancreatitis were the presence of a rare variant, GGT ≥45 U/L, maximal TG value ≥40 mmol/L, and fructose consumption ≥4% of daily energy intake. One limitation of this study is that no polygenic score has been assessed. Therefore, the proportion of patients in the negative-MCS group having a high polygenic risk as compared to those that are genetically undefined is not known (30).

In a study of 103 Chinese subjects with TG above 5.65 mmol/l and without secondary causes of HTG, patients with history of AP presented a higher frequency of rare variants in the canonical genes involved in TG metabolism than those with no history of AP. However, for several of these subjects, the variant in question was a variant of uncertain significance (36). Interestingly, the maximal TG value was also significantly different between subjects with history of AP (16.6 mmol/L) and those with no history of AP (11.3 mmol/L) (36).

## Treatment

The main goal in the treatment of MCS patients is to reduce the TG concentration below the threshold of 5.6 mmol/L (500 mg/dL) in order to prevent AP (41, 42). The secondary focus of treatment is then to reduce the cardiovascular risk. The first-line treatment for these patients is to manage secondary factors associated with HTG such as physical inactivity, obesity, metabolic syndrome, alcohol intake, and uncontrolled diabetes, as well as pharmacological treatment with fibrates. One of the mechanisms by which fibrates lower TG concentrations is by increasing LPL-mediated lipolysis. Therefore, this drug is generally effective in MCS patients, in which LPL activity is not completely impaired, but it is poorly effective in FCS patients, in which a marked reduction or complete loss of LPL activity is present. In the study of Paquette et al. including 75 MCS patients with a mean baseline TG > 10 mmol/L, fibrate use was associated with a ≥30% TG reduction in 83% of the cohort and a ≥50% TG reduction in 69% of the cohort (30). In contrast, none of the FCS patients ever achieved a TG reduction of more than 30% using fibrate (30). Despite the generally good response to fibrate therapy observed in MCS patients, the efficacy of fibrate is highly heterogeneous among these patients. Therefore, the treatment target of 5.6 mmol/L to reduce the risk of AP is not often achieved. Furthermore, even if fibrates are recommended for the treatment of severe HTG, no study has specifically demonstrated that fibrates use was associated with AP risk reduction, and thus far, clinical trials showed little or no cardiovascular benefit of adding a fibrate to statin therapy (43). In FCS patients, the principal therapeutic modality remains the very low-fat diet, in which fat should be limited to 10–30 g/day or 10–15% of total energy intake. The limited intake of long-chain fatty acids is required to reduce the formation of chylomicrons and maintain adequate TG concentrations. However, this approach is very restrictive, and compliance with such a diet over a lifetime is extremely difficult (44, 45). In MCS patients, both VLDLs and chylomicrons are present in excess in the circulation and contribute to the severe HTG phenotype. While reduction of dietary fat prevents the excessive formation of chylomicrons, reduction of simple carbohydrates is associated with reduced VLDL production by the liver (46). The best dietary approach to lower the TG concentration and to prevent AP in MCS patients is still unknown. A recent study by Fantino et al. investigated for the first time in a randomized crossover design the effect of two different diets on TG concentrations in MCS participants (47). After 3 weeks on each diet, fasting TG decreased by 55% following the low-fat diet (fat: 20%, carbohydrates: 60%) and by 48% following the low-carbohydrate diet (carbohydrates: 35%, fat: 45%), without any change in body weight or in total cholesterol, HDL-C, LDL-C, and apoB. Interestingly, in a subgroup analysis including solely subjects carrying a rare variant in the *LPL* gene (positive-MCS), a more pronounced TG decrease was observed following the low-fat diet [71% (−11.97 mmol/L)] than following a low-carbohydrate diet [59% (−8.93 mmol/L)]. Therefore, this study is clinically important since it demonstrated that MCS patients can be effectively treated by either low-fat or low-carbohydrate diets if they are closely monitored by a specialized dietician, achieving a TG decrease that is comparable to the decrease obtained with fibrates. However, this study included only 12 participants, so validation of these results in a larger cohort is required. Furthermore, it is not known whether following these diets over a long-term period would result in a decreased risk of AP (47). Importantly, in some patients, a 50% decrease in TG concentration is not sufficient to reach the treatment target of 5.6 mmol/L or for TG normalization (TG ≤ 1.7 mmol/L). Fortunately, new therapies for the treatment of hypertriglyceridemia are emerging, and some of them show promising results in patients with severe HTG. These emerging therapies include molecules targeting apoC-III (volanesorsen, AKCEA-APOCIII-LRx, and AROAPOC3) (48, 49), molecules targeting ANPTL3 (evinacumab and AROANG3) (50), and ω-3 krill oil (51).

## Conclusion and Future Directions

Recent studies have helped to better characterize MCS and the metabolic complications associated with this disease. In the past few years, differences between both chylomicronemia syndromes (FCS and MCS) have been better characterized. A novelty in our understanding of MCS is the heterogeneity in the genetic susceptibility profiles leading to distinct phenotype severity. Indeed, recent studies showed that MCS susceptibility is predominantly polygenic, rather than being caused by a single rare causal variant in the five canonical genes involved in TG metabolism. However, this latter etiology is associated with a more severe form of MCS, with increased risk of life-threatening AP. Furthermore, measurement of apoB in patients with severe HTG could be a pertinent first step to identify higher-risk individuals. Despite these new findings, the factors that explain the heterogeneity in the risk of AP in MCS patients remain poorly understood, and more studies are required in this field. It has been demonstrated that both low-fat and low-carbohydrate diets are associated with TG reduction of ~50% in these patients, allowing flexibility in the implementation of lifestyle interventions that may encourage better compliance. However, future studies in MCS patients should aim at investigating whether following these diets over a long-term period would result in a decreased risk of adverse outcomes such as AP, development of NAFLD, or cardiovascular events. In addition, the difference in the response to different diets or interventions according to the type of genetic predisposition should be investigated. This review highlights the importance of performing a genetic screening in patients with severe HTG in order to improve risk stratification and to identify potential candidates for new biologic therapies. However, several challenges surrounding the genetic characterization of these patients remain, such as the question of accessibility and cost. Furthermore, a large part of the severe HTG population is still genetically undefined or carries variants of uncertain significance. It would be interesting to investigate if the use of omnigenic scores would result in an improved genetic characterization of MCS patients.

## Author Contributions

MP and SB: writing and editing. Both authors contributed to the article and approved the submitted version.

## Conflict of Interest

SB received research grants from Akcea, Fondation Leducq and Fondation Yvan Morin. She has participated in clinical research protocols from Akcea, Amgen, Ionis Pharmaceuticals, Inc., The Medicines Company, Novartis, Pfizer and Sanofi. She has served on advisory boards for Akcea, Amgen, HLS Therapeutics, Novartis, Novo Nordisk and Sanofi, and received honoraria for symposia from Akcea, Amgen, Novo Nordisk and Sanofi. The remaining author declares that the research was conducted in the absence of any commercial or financial relationships that could be construed as a potential conflict of interest.

## Publisher's Note

All claims expressed in this article are solely those of the authors and do not necessarily represent those of their affiliated organizations, or those of the publisher, the editors and the reviewers. Any product that may be evaluated in this article, or claim that may be made by its manufacturer, is not guaranteed or endorsed by the publisher.

## Abbreviations

ALT: alanine aminotransferase
AP: acute pancreatitis
ApoB: apolipoprotein B
BMI: body mass index
CVD: cardiovascular disease
FCS: familial chylomicronemia syndrome;
GGT: gamma-glutamyl transferase
HDL-C: high-density lipoprotein cholesterol
HTG: hypertriglyceridemia
LDL-C: low-density lipoprotein cholesterol
LPL: lipoprotein lipase;
MCS: multifactorial chylomicronemia syndrome
NAFLD: nonalcoholic fatty liver disease;
SNPs: single-nucleotide polymorphisms
TG: triglyceride
VLDL: very-low-density lipoprotein.

## References

[1] Brahm A Hegele RA. Hypertriglyceridemia. Nutrients. (2013) 5:981–1001. 10.3390/nu5030981 23525082 PMC3705331

[2] Johansen CT Kathiresan S Hegele RA. Genetic determinants of plasma triglycerides. J Lipid Res. (2011) 52:189–206. 10.1194/jlr.R009720 21041806 PMC3023540

[3] Ford ES Li C Zhao G Pearson WS Mokdad AH. Hypertriglyceridemia and its pharmacologic treatment among US adults. Arch Intern Med. (2009) 169:572–8. 10.1001/archinternmed.2008.599 19307519

[4] Berberich AJ Ouédraogo AM Shariff SZ Hegele RA Clemens KK. Incidence, predictors and patterns of care of patients with very severe hypertriglyceridemia in Ontario, Canada: a population-based cohort study. Lipids Health Dis. (2021) 20:98. 10.1186/s12944-021-01517-6 34479547 PMC8417954

[5] Dron JS Wang J Cao H McIntyre AD Iacocca MA Menard JR. Severe hypertriglyceridemia is primarily polygenic. J Clin Lipidol. (2019) 13:80–8. 10.1016/j.jacl.2018.10.006 30466821

[6] Brahm AJ Hegele RA. Chylomicronaemia–current diagnosis and future therapies. Nat Rev Endocrinol. (2015) 11:352–62. 10.1038/nrendo.2015.26 25732519

[7] Tremblay K Méthot J Brisson D Gaudet D. Etiology and risk of lactescent plasma and severe hypertriglyceridemia. J Clin Lipidol. (2011) 5:37–44. 10.1016/j.jacl.2010.11.004 21262505

[8] Baass A Paquette M Bernard S Hegele RA. Familial chylomicronemia syndrome: an under-recognized cause of severe hypertriglyceridaemia. J Intern Med. (2020) 287:340–8. 10.1111/joim.13016 31840878

[9] Goldberg RB Chait A. A Comprehensive update on the chylomicronemia syndrome. Front Endocrinol. (2020) 11:593931. 10.3389/fendo.2020.593931 33193106 PMC7644836

[10] Warden BA Minnier J Duell PB Fazio S Shapiro MD. Chylomicronemia syndrome: familial or not? J Clin Lipidol. (2020) 14:201–6. 10.1016/j.jacl.2020.01.014 32107181

[11] Belhassen M Van Ganse E Nolin M Bérard M Bada H Bruckert E. 10-Year comparative follow-up of familial versus multifactorial chylomicronemia syndromes. J Clin Endocrinol Metab. (2021) 106:e1332–42. 10.1210/clinem/dgaa838 33221907

[12] Nordestgaard BG Varbo A. Triglycerides and cardiovascular disease. Lancet. (2014) 384:626–35. 10.1016/S0140-6736(14)61177-6 25131982

[13] Valdivielso P Ramírez-Bueno A Ewald N. Current knowledge of hypertriglyceridemic pancreatitis. Eur J Intern Med. (2014) 25:689–94. 10.1016/j.ejim.2014.08.008 25269432

[14] Ewald N Hardt PD Kloer HU. Severe hypertriglyceridemia and pancreatitis: presentation and management. Curr Opin Lipidol. (2009) 20:497–504. 10.1097/MOL.0b013e3283319a1d 19770656

[15] Bálint ER Fur G Kiss L Németh DI Soós A Hegyi P. Assessment of the course of acute pancreatitis in the light of aetiology: a systematic review and meta-analysis. Sci Rep. (2020) 10:17936. 10.1038/s41598-020-74943-8 33087766 PMC7578029

[16] Kiss L Fur G Mátrai P Hegyi P Ivány E Cazacu IM. The effect of serum triglyceride concentration on the outcome of acute pancreatitis: systematic review and meta-analysis. Sci Rep. (2018) 8:14096. 10.1038/s41598-018-32337-x 30237456 PMC6147944

[17] Younossi ZM Koenig AB Abdelatif D Fazel Y Henry L Wymer M. Global epidemiology of nonalcoholic fatty liver disease-Meta-analytic assessment of prevalence, incidence, and outcomes. Hepatology. (2016) 64:73–84. 10.1002/hep.28431 26707365

[18] Argo CK Caldwell SH. Epidemiology and natural history of non-alcoholic steatohepatitis. Clin Liver Dis. (2009) 13:511–31. 10.1016/j.cld.2009.07.005 19818302

[19] Marchesini G Brizi M Bianchi G Tomassetti S Bugianesi E Lenzi M. Nonalcoholic fatty liver disease: a feature of the metabolic syndrome. Diabetes. (2001) 50:1844–50. 10.2337/diabetes.50.8.1844 11473047

[20] Kotronen A Yki-Järvinen H. Fatty liver: a novel component of the metabolic syndrome. Arterioscler Thromb Vasc Biol. (2008) 28:27–38. 10.1161/ATVBAHA.107.147538 17690317

[21] Bedogni G Miglioli L Masutti F Tiribelli C Marchesini G Bellentani S. Prevalence of and risk factors for nonalcoholic fatty liver disease: the Dionysos nutrition and liver study. Hepatology. (2005) 42:44–52. 10.1002/hep.20734 15895401

[22] Maltais M Brisson D Gaudet D. Non-alcoholic fatty liver in patients with chylomicronemia. J Clin Med. (2021) 10:669. 10.3390/jcm10040669 33572376 PMC7916177

[23] Moulin P Dufour R Averna M Arca M Cefalù AB Noto D. Identification and diagnosis of patients with familial chylomicronaemia syndrome (FCS): expert panel recommendations and proposal of an “FCS score”. Atherosclerosis. (2018) 275:265–72. 10.1016/j.atherosclerosis.2018.06.814 29980054

[24] Paquette M Bernard S Hegele RA Baass A. Chylomicronemia: differences between familial chylomicronemia syndrome and multifactorial chylomicronemia. Atherosclerosis. (2019) 283:137–42. 10.1016/j.atherosclerosis.2018.12.019 30655019

[25] D'Erasmo L Di Costanzo A Cassandra F Minicocci I Polito L Montali A. Spectrum of mutations and long-term clinical outcomes in genetic chylomicronemia syndromes. Arterioscler Thromb Vasc Biol. (2019) 39:2531–41. 10.1161/ATVBAHA.119.313401 31619059

[26] O'Dea LSL MacDougall J Alexander VJ Digenio A Hubbard B Arca M. Differentiating familial chylomicronemia syndrome from multifactorial severe hypertriglyceridemia by clinical profiles. J Endocr Soc. (2019) 3:2397–410. 10.1210/js.2019-00214 31777768 PMC6864364

[27] Rioja J Ariza MJ García-Casares N Coca-Prieto I Arrobas T Muñiz-Grijalvo O. Evaluation of the chylomicron-TG to VLDL-TG ratio for type I hyperlipoproteinemia diagnostic. Eur J Clin Invest. (2020) 50:e13345. 10.1111/eci.13345 32649781

[28] Tremblay K Gaudet D Khoury E Brisson D. Dissection of clinical and gene expression signatures of familial versus multifactorial chylomicronemia. J Endocr Soc. (2020) 4:bvaa056. 10.1210/jendso/bvaa056 32537545 PMC7278277

[29] Ariza MJ Rioja J Ibarretxe D Camacho A Díaz-Díaz JL Mangas A. Molecular basis of the familial chylomicronemia syndrome in patients from the national dyslipidemia registry of the spanish atherosclerosis society. J Clin Lipidol. (2018) 12:1482–92.e3. 10.1016/j.jacl.2018.07.013 30150141

[30] Paquette M Amyot J Fantino M Baass A Bernard S. Rare variants in triglycerides-related genes increase pancreatitis risk in multifactorial chylomicronemia syndrome. J Clin Endocrinol Metab. (2021) 106:e3473–82. 10.1210/clinem/dgab360 34019660

[31] de Graaf J Couture P Sniderman A. A diagnostic algorithm for the atherogenic apolipoprotein B dyslipoproteinemias. Nat Clin Pract Endocrinol Metab. (2008) 4:608–18. 10.1038/ncpendmet0982 18838971

[32] Hegele RA Dron JS. 2019 George lyman duff memorial lecture: three decades of examining DNA in patients with dyslipidemia. Arterioscler Thromb Vasc Biol. (2020) 40:1970–81. 10.1161/ATVBAHA.120.313065 32762461

[33] Dron JS Dilliott AA Lawson A McIntyre AD Davis BD Wang J. Loss-of-function CREB3L3 variants in patients with severe hypertriglyceridemia. Arterioscler Thromb Vasc Biol. (2020) 40:1935–41. 10.1161/ATVBAHA.120.314168 32580631

[34] Glodowski M Christen S Saxon DR Hegele RA Eckel RH. Novel PPARG mutation in multiple family members with chylomicronemia. J Clin Lipidol. (2021) 15:431–4. 10.1016/j.jacl.2021.03.006 33832869

[35] Gill PK Dron JS Hegele RA. Genetics of hypertriglyceridemia and atherosclerosis. Curr Opin Cardiol. (2021) 36:264–71. 10.1097/HCO.0000000000000839 33818545

[36] Jin JL Sun D Cao YX Zhang HW Guo YL Wu NQ. Intensive genetic analysis for Chinese patients with very high triglyceride levels: Relations of mutations to triglyceride levels and acute pancreatitis. EBioMedicine. (2018) 38:171–7. 10.1016/j.ebiom.2018.11.001 30420299 PMC6306308

[37] Paquette M Baass A. Polygenic risk scores for cardiovascular disease prediction in the clinical practice: are we there? Atherosclerosis. (2022) 340:46–7. 10.1016/j.atherosclerosis.2021.11.030 34895918

[38] Dron JS Hegele RA. The evolution of genetic-based risk scores for lipids and cardiovascular disease. Curr Opin Lipidol. (2019) 30:71–81. 10.1097/MOL.0000000000000576 30676533

[39] Gill PK Dron JS Dilliott AA McIntyre AD Cao H Wang J. Ancestry-specific profiles of genetic determinants of severe hypertriglyceridemia. J Clin Lipidol. (2021) 15:88–96. 10.1016/j.jacl.2020.11.007 33303403

[40] Gagné C Brun LD Julien P Moorjani S Lupien PJ. Primary lipoprotein-lipase-activity deficiency: clinical investigation of a French Canadian population. CMAJ. (1989) 140:405–11. 2914262 PMC1268664

[41] Scherer J Singh VP Pitchumoni CS Yadav D. Issues in hypertriglyceridemic pancreatitis: an update. J Clin Gastroenterol. (2014) 48:195–203. 10.1097/01.mcg.0000436438.60145.5a 24172179 PMC3939000

[42] Christian JB Arondekar B Buysman EK Johnson SL Seeger JD Jacobson TA. Clinical and economic benefits observed when follow-up triglyceride levels are less than 500 mg/dL in patients with severe hypertriglyceridemia. J Clin Lipidol. (2012) 6:450–61. 10.1016/j.jacl.2012.08.007 23009781

[43] Ginsberg HN Elam MB Lovato LC Crouse JR 3rd Leiter LA Linz P. Effects of combination lipid therapy in type 2 diabetes mellitus. N Engl J Med. (2010) 362:1563–74. 10.1056/NEJMoa1001282 20228404 PMC2879499

[44] Whitfield-Brown L Hamer O Ellahi B Burden S Durrington P. An investigation to determine the nutritional adequacy and individuals experience of a very low fat diet used to treat type V hypertriglyceridaemia. J Hum Nutr Diet. (2009) 22:232–8. 10.1111/j.1365-277X.2009.00945.x 19504738

[45] Davidson M Stevenson M Hsieh A Ahmad Z Roeters van Lennep J Crowson C. The burden of familial chylomicronemia syndrome: results from the global IN-FOCUS study. J Clin Lipidol. (2018) 12:898–907.e2. 10.1016/j.jacl.2018.04.009 29784572

[46] Parks EJ. Effect of dietary carbohydrate on triglyceride metabolism in humans. J Nutr. (2001) 131:2772S−4. 10.1093/jn/131.10.2772S 11584104

[47] Fantino M Paquette M Blais C Saint-Pierre N Bourque L Baass A. Both low-fat and low-carbohydrate diets reduce triglyceride concentration in subjects with multifactorial chylomicronemia syndrome: a randomized crossover study. Nutr Res. (2022) 101:43–52. 10.1016/j.nutres.2022.02.001 35367870

[48] Gouni-Berthold I Alexander VJ Yang Q Hurh E Steinhagen-Thiessen E Moriarty PM. Efficacy and safety of volanesorsen in patients with multifactorial chylomicronaemia (COMPASS): a multicentre, double-blind, randomised, placebo-controlled, phase 3 trial. Lancet Diabetes Endocrinol. (2021) 9:264–75. 10.1016/S2213-8587(21)00046-2 33798466

[49] D'Erasmo L Gallo A Di Costanzo A Bruckert E Arca M. Evaluation of efficacy and safety of antisense inhibition of apolipoprotein C-III with volanesorsen in patients with severe hypertriglyceridemia. Expert Opin Pharmacother. (2020) 21:1675–84. 10.1080/14656566.2020.1787380 32646313

[50] Ahmad Z Pordy R Rader DJ Gaudet D Ali S Gonzaga-Jauregui C. Inhibition of angiopoietin-like protein 3 with evinacumab in subjects with high and severe hypertriglyceridemia. J Am Coll Cardiol. (2021) 78:193–5. 10.1016/j.jacc.2021.04.091 34238441

[51] Mozaffarian D Maki KC Bays HE Aguilera F Gould G Hegele RA. Effectiveness of a novel ω-3 krill oil agent in patients with severe hypertriglyceridemia: a randomized clinical trial. JAMA Netw Open. (2022) 5:e2141898. 10.1001/jamanetworkopen.2021.41898 34989797 PMC8739762

